# Developmental biomechanics and age polyethism in leaf-cutter ants

**DOI:** 10.1098/rspb.2023.0355

**Published:** 2023-06-14

**Authors:** Frederik Püffel, Lara Meyer, Natalie Imirzian, Flavio Roces, Richard Johnston, David Labonte

**Affiliations:** ^1^ Department of Bioengineering, Imperial College London, London, UK; ^2^ Faculty of Nature and Engineering, City University of Applied Sciences Bremen, Bremen, Germany; ^3^ Department of Behavioural Physiology and Sociobiology, University of Würzburg, Würzburg, Germany; ^4^ Materials Research Centre, Swansea University, Swansea, UK

**Keywords:** division of labour, social insects, bite forces, behavioural development

## Abstract

Many social insects display age polyethism: young workers stay inside the nest, and only older workers forage. This behavioural transition is accompanied by genetic and physiological changes, but the mechanistic origin of it remains unclear. To investigate if the mechanical demands on the musculoskeletal system effectively prevent young workers from foraging, we studied the biomechanical development of the bite apparatus in *Atta vollenweideri* leaf-cutter ants. Fully matured foragers generated peak *in vivo* bite forces of around 100 mN, more than one order of magnitude in excess of those measured for freshly eclosed callows of the same size. This change in bite force was accompanied by a sixfold increase in the volume of the mandible closer muscle, and by a substantial increase of the flexural rigidity of the head capsule, driven by a significant increase in both average thickness and indentation modulus of the head capsule cuticle. Consequently, callows lack the muscle force capacity required for leaf-cutting, and their head capsule is so compliant that large muscle forces would be likely to cause damaging deformations. On the basis of these results, we speculate that continued biomechanical development post eclosion may be a key factor underlying age polyethism, wherever foraging is associated with substantial mechanical demands.

## 1. Introduction

Social insects are extremely ‘successful’ [1,2], and this success is thought to be partially based on the evolution of a ‘division of labour’; some tasks are preferentially or exclusively performed by specific individuals. In many social insects, such task preferences transcend the elementary dichotomy between reproductive and non-reproductive labour, and sterile workers show preferences and specialization for subsets of non-reproductive colony tasks. The classic explanation for this phenomenon suggests that task specialization increases the ergonomic efficiency of colonies, and thus their fitness (e.g. [3], but see [4]). Two main themes are common to studies which propose explanations for the benefits *of* or possible mechanisms *for* the evolution of a non-reproductive division of labour (e.g. [5–8]): task preferences are associated with differences in *worker phenotype*, for example in terms of worker size or body shape, or with differences in *worker age*.

In social insects, systematic changes in task preferences with age, or age polyethism, have evolved in bees [9–13], wasps [14,15], ants [16–25] and termites [26]: freshly eclosed workers tend to stay inside the nest and attend to queen and brood, and only older workers engage in foraging tasks outside the nest. This behavioural transition typically occurs within the first few weeks after eclosion (e.g. [9,22,23,26]), although the exact timeline is subject to variation based on genotype [20] and colony size [21,27].

Because of its frequent emergence and importance to the ecology of social insects, age polyethism has received considerable attention from behavioural biologists [13,15,20,21,27], ecologists [14,28,29], geneticists [30,31], neuroethologists [22,32,33] and biomechanists alike [34,35], and several genetic and physiological correlates have been identified. For example, the transition from within-nest to outside foraging tasks is accompanied by substantial changes in gene expression [30,31,36], hormone and neuropeptide levels [14,15,27,37], the exocrine system [13], muscle chemistry [38–40] and brain physiology and size [33,41].

In particular, the physiological development following eclosion is hypothesised to be key in determining the ability of workers to perform specific tasks [7,12,22,24,32], suggesting an emergence of age polyethism based on the acquisition of new capabilities [22] (but see [6,8]). However, another factor that may influence worker capabilities has received considerably less attention: the biomechanical development after eclosion [32,34,35,42]. Biomechanical factors are likely relevant, because outside foraging tasks are associated with substantial mechanical demands: biting, piercing, sucking and material transportation by flight or terrestrial locomotion all require sufficiently large muscle forces and a robust skeleton to transmit these forces without inflicting damage. To investigate how muscle forces and skeletal rigidity change in the days after eclosion, we here conduct a study of the biomechanical development of a musculoskeletal apparatus that is of particular importance in foraging, in a social insect where the mechanical demands on it are particularly strong: the head capsule of leaf-cutter ants.

**Figure 1.**
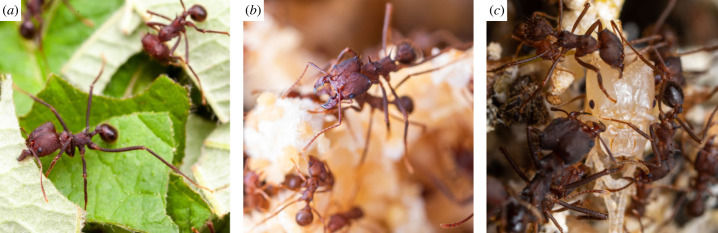
In leaf-cutter ant colonies, foraging tasks are only performed by workers exceeding a minimum age after eclosion (*a*), younger workers stay inside the nest and care for fungus (*b*) and pupae (*c*). To investigate if this behavioural transition may be driven by a change in mechanical ability to forage, we investigated the biomechanical development of the musculoskeletal bite apparatus from freshly eclosed callows to fully matured foragers. Photo credit: Samuel T. Fabian.

Leaf-cutter ants forage by cutting and transporting leaf fragments from fresh vegetation surrounding the nest [43,44] (figure 1). To meet the high mechanical demands of plant cutting, excessively large weight-specific bite forces have evolved in leaf-cutter ants [45], and leaf-cutting consequently involves a metabolic scope close to that measured for insect flight [46]. The ability to partake in foraging thus depends at least partly on the physiology of the mandible closer and opener muscles, and the mechanical robustness of the skeletal system. Previous work suggested that the musculoskeletal bite apparatus of ants may not have fully matured at the time of eclosion: tooth hardness of leaf-cutter ant mandibles increases nearly threefold in the days after eclosion, a development correlated with corresponding changes in zinc-concentration [34]; and the cephalic muscles of *Pheidole* ants grow substantially post eclosion [32]. Both results suggest the possibility of age-related constraints on foraging ability based on cuticle and muscle development.

Our study builds on this work by quantifying the biomechanical development of the musculoskeletal bite apparatus of *Atta vollenweideri* leaf-cutter ants; we investigated to which extent mechanical ability varies in the days following eclosion by (i) measuring peak voluntary bite forces with a custom-built force setup, (ii) quantifying the volume of the mandible closer muscle and the thickness of the head capsule using μCT imaging, and (iii) extracting the indentation modulus of the cuticle via nanoindentation experiments. By establishing a biomechanical paradigm to investigate age-related changes in the ability to partake in foraging tasks, we hope to increase our understanding of the physical constraints that may contribute to age polyethism in insects.

## 2. Material and methods

### (a) Study animals

We used workers from three *Atta vollenweideri* leaf-cutter ant colonies (‘*E*’, ‘*D*’ and ‘*F*’), all founded and collected in Uruguay in 2014. The colonies were fed with bramble, cornflakes and honey water *ad libitum*, and kept under a 12 h : 12 h light : dark cycle at $25^{\circ }C$ and 50% humidity in a climate chamber. In order to minimize confounding effects due to worker size differences [45,47], we only collected small workers with a body mass between 3 and 7 mg, representing the lower end of forager sizes in *A. vollenweideri* (2.5–26.9 mg, see [48]). We collected 49 ants: 14 fully darkened workers from the foraging area (*n*_*F*_ = 14), and 35 callows of varying cuticle brightness from the fungal gardens (*n*_*D*_ = 7, *n*_*E*_ = 8, *n*_*F*_ = 20). Callows were extracted by scooping fresh fungus from boxes that appeared to contain a large amount of brood into a separate container, from which those ants with visibly brighter cuticle were carefully extracted using insect tweezers. We selected ants such that there was no significant difference in mean body mass between callows (4.5 ± 1.2 mg) and foragers (4.8 ± 1.2 mg; Wilcoxon rank sum test: *W* = 214.5, *p* = 0.51). To test if body mass increases during maturation as a result of tissue growth (see results), which would render a size-independent comparison between callows and foragers difficult, we extracted a second size-metric from tomographic scans (see below). We measured the distance between the mandibular joint centres (as defined in [47])—a metric we considered unlikely to change significantly with age. For callows, the joint centre-to-centre distance was 1.13 ± 0.12 mm, not significantly different to that of foragers (1.20 ± 0.17 mm, two-sample *t*-test: *t*_8_ = −0.78, *p* = 0.46). We hence assume that changes in body mass during maturation are negligible. We combined data from all three colonies as previous work demonstrated that bite forces are consistent across *A. vollenweideri* colonies [45].

### (b) Bite force measurements

**Figure 2.**
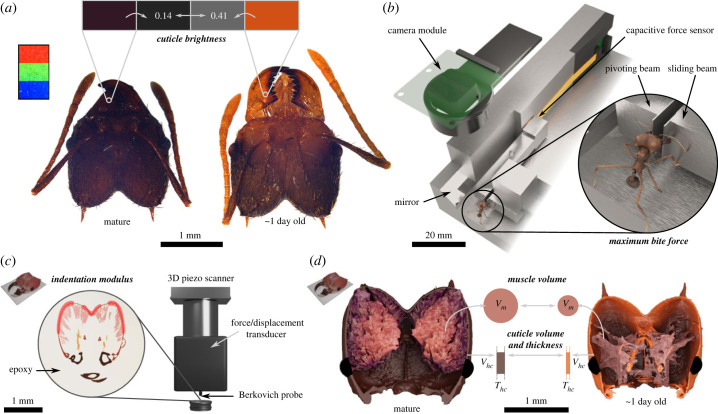
In order to study the biomechanical development of the musculoskeletal bite apparatus post eclosion, we extracted adult *Atta vollenweideri* leaf-cutter ants at different time points after eclosion from the foraging area and fungal gardens of three different colonies. We quantified *cuticle brightness*, *maximum bite force*, *indentation modulus*, *muscle volume* and *cuticle volume & thickness*. (*a*) As proxy for age post eclosion, we measured the brightness of the mandible cuticle from standardized photographs, both for randomly selected ants, and for a smaller subset of ants of known age after eclosion. (*b*) We quantified bite performance with a custom-built force sensor (described in detail in [45], three-dimensional model of biting ant created by Fabian Plum with the open-source photogrammetry platform ‘scAnt’; see [49]). (*c*,*d*) In order to assess the change in structural rigidity of the head capsule, we measured its indentation modulus via nanoindentation experiments at several locations in the horizontal head plane (red areas), and its average thickness, *T*_*hc*_, from *μ*CT images. From these images, we also extracted the volumes of the mandible closer muscle and head cuticle, *V*_*m*_ and *V*_*hc*_, respectively.

In order to quantify bite performance, we measured the maximum voluntary bite force using a custom-built set-up described in detail in Püffel *et al.* [45]. In brief, individual ants were held in front of two bite plates using insect tweezers (figure 2*b*). The ants then readily bit onto the two bite plates, both protruding from two mechanically uncoupled beams. The first beam can freely rotate about a pivot and then pushes onto a capacitive force sensor, which is thus compressed when a force is applied to the bite plate. The second beam remains stationary, such that the distance between the two outer surfaces of both bite plates—the mandibular gape required to bite—is approximately constant at 0.5 mm, or roughly a third of the average head width. Each measurement was terminated after at least five complete bite cycles or a total bite duration of more than 10 s. For the youngest and supposedly weakest callows, this condition was difficult to verify from the force trace alone, as peak bite forces were below 10 mN, equivalent to about twice the sensor noise (see results). For these ants (*n* = 11), we identified bites based on direct observations via a top-down camera, which filmed the ants during the experiment with 30 fps. After the bite force experiment, all ants were weighed (AX304 Microbalance, 310 g × 0.1 mg, Mettler Toledo, Greifensee, Switzerland), and sacrificed by freezing.

From the recorded bite force traces, the maximum bite forces were extracted. However, these force maxima are still influenced by head orientation and the location of the point of force transmission between the mandible and bite plate. In order to remove the influence of these factors, we calculated the maximum bite force at an equivalent mandible outlever, the most distal point of the mandible, using the procedure described in detail in Püffel *et al.* [45]. We did not correct for differences in mandibular opening angle as this requires assumptions on muscle physiology [45]. However, the opening angles of callow and forager bites were almost identical, $78\pm 6^{\circ }$ versus $77\pm 5^{\circ }$ (two sample *t*-test: *t*_47_ = 0.63, *p* = 0.53), so that any effects are likely unsystematic. Bite forces of mature *A. vollenweideri* ants are maximal at an opening angle of about $60^{\circ }$; the forces measured here are about 15% lower than this peak [45].

### (c) Cuticle brightness

In order to approximate the age of the collected workers post eclosion, we measured cuticle brightness (e.g. [16,22,34]), which typically decreases after eclosion, in concert with cuticle sclerotization ([50], and see below). To make this qualitative link quantitative, we extracted the cuticle brightness from an additional set of nine callows of known age post eclosion (also see [32]). To this end, late-stage pupae were taken from the fungal gardens and placed in a separate container (≈15 × 8 × 5 cm) with a small amount of fungus and dozens of minims to maintain them. The pupae were checked daily, and as soon as the legs had unfolded, they were marked with paint (Edding 4000 paint marker, Edding AG, Ahrensburg, Germany; see [42]), and sacrificed after one (*n* = 3, 4.1 ± 0.9 mg), three (*n* = 3, 4.1 ± 0.8 mg) or five further days in the container (*n* = 3, 4.5 ± 1.0 mg; the difference in body mass between these subsets was not significant, ANOVA: *F*_1,6_ = 0.22, *p* = 0.81).

To quantify cuticle brightness, we photographed worker head capsules in the horizontal plane using a high-resolution light microscope equipped with an apochromatic lens (camera: DMC5400, microscope: Z6 Apo controlled via *LAS X*; Leica Microsystems GmbH, Wetzlar, Germany; figure 2*a*). Heads were placed on white paper next to a printed RGB colour stripe, which served as a baseline to enable comparison between relative colour differences across photographs (figure 2*a*). To minimize such differences, we used the same exposure time and colour profile in *LAS X*, and kept lighting conditions approximately constant, using the microscope-internal light source and two external LED lamps. From each image, the RGB values for a defined set of ‘regions of interest’ (ROIs) were extracted using the ‘Colour histogram’ function in *Fiji* [51]. A rectangular ROI was taken from each colour stripe (red, green, blue), approximately spanning 200 × 150 pixels, and one circular ROI from a ‘tooth-free’ region of the mandible blade with a diameter of 30 pixels (figure 2*a*).

The perceived colour of the mandible is probably affected by both pigmentation and variations in translucency of the cuticle due to local variations in thickness. To minimize confounding effects, we always measured cuticle brightness at locations where the left and right mandible did not overlap. We then calculated cuticle brightness from the RGB values as *b*_RGB_ = (*R* + *G* + *B*)/(3 · 255), equivalent to the unweighted greyscale conversion native to *Fiji*.

Neither body mass nor the brightness of the colour stripe differed significantly between monitored callows (*n* = 9) and all other workers (*n* = 49; Wilcoxon rank sum test, body mass: *W* = 197.5, *p* = 0.63; brightness: *W* = 374, *p* = 0.18). However, we found a significant negative correlation between body mass and cuticle brightness across the ants used for bite experiments (Spearman’s rank correlation: *ρ*_47_ = −0.38, *p* < 0.01), such that dark ants (*b*_RGB_ < 0.20) weighed 5.2 ± 1.2 mg, and bright ants (*b*_RGB_ > 0.35) weighed 4.2 ± 1.2 mg, or approximately 20% less. This effect may be attributed to size-dependent differences in mandible thickness, which resulted in age-independent differences in cuticle translucency and brightness. We however argue that any confounding effects of body mass are small in comparison to the effects of ageing and the associated development of the bite apparatus (see below).

### (d) Nanoindentation

To investigate if the material properties of the head capsule change in the days post eclosion, we conducted nanoindentation experiments; this technique involves pushing a small probe with well-defined geometry into a material while simultaneously recording both force and displacement. Material properties such as the indentation modulus and indentation hardness can then be extracted from the relationship between force and displacement, using established mechanical theory [52]. We used a subset of 12 ants from the 49 biting ants for the experiments, selected to cover the range of observed cuticle brightness: eight callows (4.3 ± 1.2 mg) and four foragers (4.3 ± 1.2 mg; the difference in body mass was not significant, Two sample *t*-test: *t*_10_ = 0.03, *p* = 0.97). To expose a smooth cross-section of the head capsule, the samples were first embedded in two-part epoxy (EPO-Set, MetPrep Ltd, Coventry, UK), so that the dorsal head plane faced upwards (figure 2*c*). To facilitate head capsule alignment, an insect pin was pierced into each head capsule prior to embedding. After curing for at least 6 h, the samples were then ground (Saphir 250 A2-ECO, QATM, Mammelzen, Germany) using abrasive silicon–carbide paper of increasing grit numbers (400, 800, 1200, 2500, 4000) in single pressure mode at 25 N for 30, 90, 120, 180 and 180 s, respectively. All samples were then polished with 0.3 μm alumina and 0.06 μm colloidal silica suspensions (MetPrep Ltd, Coventry, UK), at 15 N for 120 s each.

Indentations were performed with a Hysitron TriboIndenter (Ti 950, Bruker Corporation, Billerica, MA, USA) and a Berkovich probe (a three-sided pyramid manufactured from diamond). Each sample was indented in a single cross-sectional plane, but numerous times (19 ± 9), and in three different regions where the closer muscle attaches (see red areas in figure 2*c*). The minimum distance between indents was approximately 20 μm; all indents were at least ≈2 μm away from the cuticle–epoxy interface. We used a trapezoidal loading profile in closed-loop displacement control, with a load time of 5 s, a peak displacement of 300 nm, a hold time of 20 s, and an unloading time of 5 s. The indentation modulus was extracted from the unloading curve using the native control software and Oliver–Pharr analysis [53]; the tip-area function of the Berkovich tip was calibrated with 100 indents on fused quartz, and confirmed on polycarbonate standards supplied by the manufacturer. All measurements were conducted at ambient conditions.

### (e) Tomography and morphometric analysis

In order to quantify muscle volume and the thickness of the head capsule, an additional subset of 10 ants was prepared for *μ*CT imaging: seven callows (4.2 ± 1.3 mg) and three foragers (4.7 ± 1.5 mg); the difference in body mass was not significant (Two sample *t*-test: *t*_8_ = −0.60, *p* = 0.56). For mature workers, the labrum and antennae were removed with forceps, and about five additional holes were pierced into the head capsule using insect pins to facilitate fixative penetration. For the callows, only the antennae were removed, and the head capsule was otherwise left intact; this precaution was necessary to minimize deformation of the head capsule during manual manipulation (see discussion). All heads were fixed in paraformaldehyde solution (4% in PBS, Thermo Fisher Scientific, Waltham, MA, USA) for 18 h, and subsequently dehydrated via storage in 70%, 80%, 90% and 100% ethanol for 1 h each.

Prior to scanning, the samples were stained with 1% iodine in ethanol for 48–168 h [54], rinsed, and transferred to a pipette tip with 95% ethanol (for more details, see electronic supplementary material). The samples were imaged via X-ray microscopy (XRM), using a laboratory-based Xradia Versa 520 (Carl Zeiss XRM Inc., Dublin, CA, USA, with a tube voltage of 70 kV, current of 85 μA, and exposure time of 500 ms), a CCD detector system with scintillator-coupled visible light optics, and a tungsten transmission target. A low energy filter was placed in the beam path (LE1, proprietary Carl Zeiss microscopy filter), and a total of 2401 projections were captured at a 4× lens magnification with 2× binning over a ‘180 degrees plus fan angle’ range. The tomograms were reconstructed from two-dimensional projections using a commercial software package (XMReconstructor, Carl Zeiss XRM Inc., Dublin, CA, USA), with a cone-beam reconstruction algorithm based on filtered back-projection, resulting in 8-bit greyscale image stacks with isotropic voxel sizes between 2.6 and 3.4 μm, or about 15% of the smallest average head capsule thickness (see Results).

From the tomographic scans, the mandible closer muscle and head capsule were segmented in ITKSNAP (v. 3.6) [55]. The respective tissue volumes were directly exported from the software (figure 2*d*). The average cuticle thickness was obtained via the ‘LocalThickness’ function (boneJ plugin in *Fiji* [56]), performed on the image stack of the head capsule segmentation. To quantify the error of the thickness measurement, we created an artificial three-dimensional image of a hollow cylinder in python (v. 3.9.7) [57], with a uniform shell thickness of 10 px, within the range of average values extracted in this study (5–12 px). The estimated shell thickness was virtually uniform across the cylinder, and within ≈1% of its true value. However, a second source of error may arise from partial volume effects of the tomographic images; these effects occur at the interfaces between two materials when a voxel is partially filled by both, resulting in an intermediate greyscale value [58]. Such effects may be particularly problematic, because the lowest measured cuticle thickness was only 5 px. During segmentation, we tried to avoid the ‘smearing out’ of tissue by excluding the voxels that had greyscale values closer to those of the tissue surroundings (also see [59]). However, this process is subjective and its accuracy is difficult to quantify. We hence offer a second argument in support of the accuracy of our measurements and thus of the conclusions we draw from them: We photographed the head cross-sections used for nanoindentation with the microscope and camera system internal to the Hysitron TriboIndenter. We then measured cuticle thickness at 30 different locations across the sample (see red areas in figure 2*c*), as the length of the shortest line connecting both tissue boundaries. The extracted cuticle thickness of dark foragers exceeded that of the brightest callows (*b*_RGB_ > 0.35) by a factor of 2.3 (14.3 ± 2.9 μm versus 6.2 ± 1.9 μm), close to the ratio obtained from the segmented tomography scans (1.9, see Results). We note that the thickness measured using light microscopy was generally lower than the average value obtained via tomography, which was ‘biased’ upwards by thickened regions around the mandible articulation (see electronic supplementary material, figure S1*f*,*g*).

### (f) Statistical analysis

To test for significant correlation between cuticle brightness and the other experimental quantities, we performed Spearman’s rank correlation tests to account for non-normality of brightness values (Shapiro–Wilk normality test: *W*_48_ = 0.92, *p* < 0.01). Owing to methodological limitations, data for indentation modulus and cuticle thickness were unpaired and only available for a small subset of ants. To estimate both for all ants used in bite experiments, we characterized the relationship between indentation modulus, head capsule thickness and cuticle brightness via ordinary least squares (OLS) regressions on log_10_-transformed data, and then used these regressions to estimate missing values; log-transformation was necessary to meet all assumptions of the linear model [60].

## 3. Results and discussion

The behavioural transition from within-nest to outside-foraging tasks with age is well established in social insects (e.g. [9,17,22]), but it remains unclear *why* it occurs. From a biomechanical perspective, foraging requires the ability to generate and withstand substantial forces, be it for object grasping, transport, or mechanical processing. However, the development of biomechanical traits is rarely considered in the context of age polyethism (e.g. [32,34,35]). To test if the mechanical demands of foraging prevent young workers to partake in it, we quantified a set of key performance parameters of the musculoskeletal bite apparatus of *A. vollenweideri* leaf-cutter ants. Bite forces of fully matured foragers exceeded those of freshly eclosed callows by more than one order of magnitude. This variation may arise because the mandible closer muscle is not yet fully developed right after eclosion, or because the mechanical stability of the head capsule limits the maximum force that can be applied to it without causing critical deformation. In the following paragraphs, we discuss evidence for both hypotheses, and embed our findings in the context of age-related foraging in leaf-cutter ants.

### (a) Co-development of bite force and muscle post eclosion

**Figure 3.**
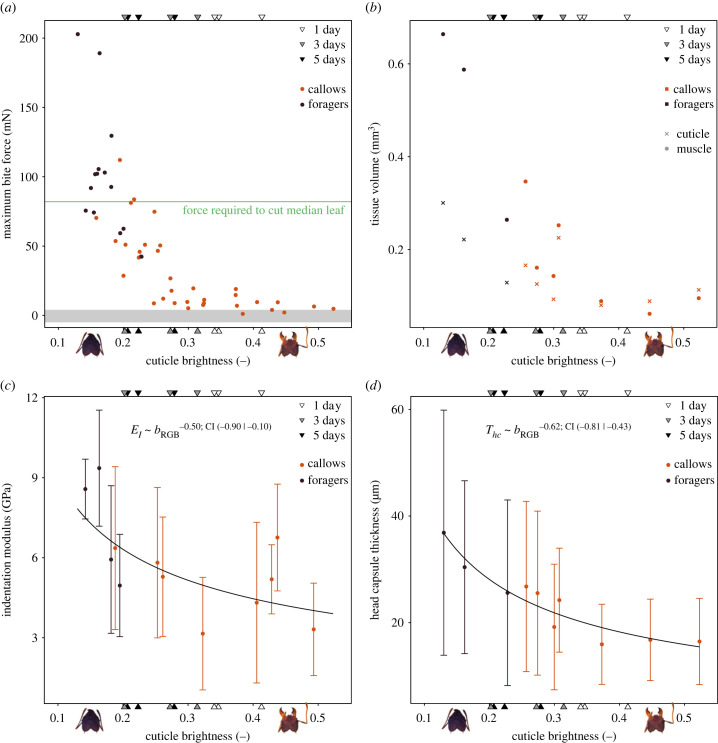
*Atta vollenweideri* leaf-cutter ant workers with varying cuticle brightness were extracted from the colonies; workers with bright or dark cuticle were extracted from the fungal gardens (callows), or from the foraging area (foragers), respectively. To link brightness to age, a set of nine late-stage pupae were photographed 1, 3 or 5 days post eclosion (triangles). (*a*) Maximum bite force negatively correlates with cuticle brightness, and decreased steeply from a maximum of more than 100 mN for workers with dark cuticle to a minimum of less than 10 mN for bright callows, just in excess of sensor noise (shaded area, *n* = 49). The poor bite performance of callows likely constrains their ability to partake in foraging activities such as leaf-cutting; the force required to cut the median tropical leaf is about 80 mN (leaf data extracted from [61]), a factor of 10 larger than the maximum bite forces callows can produce. (*b*) In order to investigate the origin of the variation in bite force, the total volume of the mandible closer muscle (circles), and of the head capsule cuticle (crosses) were extracted from segmented tomography scans. Muscle volume increased significantly by a factor of six, and cuticle volume by a factor of two between the brightest callows and fully matured workers (*n* = 10). (*c*) To determine the flexural rigidity of the head capsule, we measured both indentation modulus of the cuticle and its thickness. The indentation modulus, *E*_*I*_, decreased significantly with cuticle brightness, *b*_RGB_, by a factor of 1.5 (*n* = 12; OLS regression on log_10_-transformed data, slope: −0.50, 95% CI: [−0.90 | −0.10], *R*^2^ = 0.43). (*d*) The average head capsule thickness, *T*_*hc*_, increased from ≈15 μm for the brightest callows to ≈30 μm for foragers (*n* = 10; OLS regression on log_10_-transformed data, slope: −0.62, 95% CI: [−0.81 | −0.43], *R*^2^ = 0.87); the similar relative increase in cuticle volume and thickness suggests that most of the variation in volume stems from changes in thickness.

One key performance metric of the musculoskeletal bite apparatus is the maximum force it can generate. Across workers with different cuticle brightness, maximum voluntary bite force varied by a factor of 13, from 102 ± 46 mN for dark foragers (*b*_RGB_ = 0.17 ± 0.03) to only 8 ± 6 mN for the brightest callows (*b*_RGB_ > 0.35; figure 3*a*), barely exceeding the sensor noise. The cuticle brightness of these ants ranged from a minimum of 0.13 to a maximum of 0.52. For comparison, the cuticle brightness of the monitored ants with known age decreased significantly from 0.37 ± 0.04 at 1 day to 0.24 ± 0.04 at 5 days post eclosion (ANOVA: *F*_1,6_ = 6.84, *p* < 0.05; figure 3). These results suggest that the youngest biting callows were presumably younger than 24 h, and fully darkened foragers were older than 5 days, which may be a conservative estimate given that closely related *A. sexdens rubipilosa* ants spend three to four weeks in the callow phase [34].

**Figure 4.**
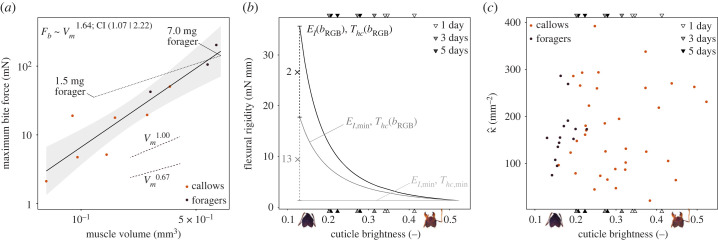
(*a*) From callows to fully darkened foragers of the same size, the maximum bite force increased with muscle volume as $F_{b}\propto {V_{m}}^{1.64}$ (OLS regression on log_10_-transformed data, 95% CI slope: [1.07 | 2.22], *R*^2^ = 0.85). This positive allometry suggests that both muscle volume and volume-specific bite force increase substantially post eclosion. For comparison, a fully matured worker of 1.5 mg has the same muscle volume at a third of the body weight of a 4.5 mg callow, and produces four times higher bite forces (*F*_*b*_ was extracted for the same opening angle as this study, [45,47]). (*b*) Muscle development is accompanied by changes in cuticle thickness and indentation modulus, leading to an increase of the flexural rigidity of the head capsule, *D*, by a factor 27 (black line). The majority of this increase stems from an increase in cuticle thickness, *T*_*hc*_ (factor 13.4), which enters the calculation of *D* as a cube (see equation (3.1)); the indentation modulus, *E*_*I*_, in contrast contributes only linearly (factor 2.0). To visualize these relative contributions, the flexural rigidity is shown (i) as the original relationship with cuticle brightness based on the regression results for both indentation modulus and thickness (*E*_*I*_(*b*_RGB_), *T*_*hc*_(*b*_RGB_), black line); (ii) for a variable thickness, *T*_*hc*_(*b*_RGB_), but constant indentation modulus, *E*_*I*,min_, fixed at its minimum (dark grey); and (iii), for constant minimum values of both indentation modulus and (*E*_*I*,min_, *T*_*hc*,min_, light grey). The cuticle brightness of freshly eclosed ants of known age is shown at the top and bottom abscissa for reference (triangles). (*c*) The ratio between maximum bite force and flexural rigidity normalized with cuticle thickness, $\hat{\kappa }$, is a proxy for cuticle strain. $\hat{\kappa }$ was not significantly correlated with cuticle brightness (*p* = 0.89; table 1), indicating an approximately constant mechanical demand on the head capsule during biting across the biomechanical development post eclosion.

The change in bite force is associated with a substantial increase in the volume of the mandible closer muscle; from bright callows (*b*_RGB_ > 0.35) to foragers, muscle volume increased by a factor of six from 0.08 ± 0.02 mm^3^ to 0.51 ± 0.21 mm^3^ (see table 1 and figure 3*b*; muscle volume was combined for both head hemispheres). To put this change into perspective, the muscle volume of callows is about equal to that of fully matured workers which are four times lighter (1.1 mg, see [47], and figure 4*a*); a size that would typically not engage in leaf-cutting [48].

**Table 1.** Results of Spearman’s rank correlation on a set of biomechanical parameters paired with cuticle brightness, *b*_RGB_. The degrees of freedom (d.f. = *n* − 2), correlation coefficients (*ρ*) and *p*-values are provided for each test. All parameters apart from $\hat{\kappa }$ were significantly negatively correlated with cuticle brightness; $\hat{\kappa }$ is a proxy for cuticle strain based on maximum bite force, and the flexural rigidity and thickness of the head capsule (see main text for details).

parameter	symbol	d.f.	*ρ*	*p*-value
maximum bite force	*F*_*b*_	47	−0.89	<0.001
muscle volume	*V*_*m*_	8	−0.92	<0.001
cuticle volume	*V*_*hc*_	8	−0.71	<0.05
cuticle thickness	*T*_*hc*_	8	−0.94	<0.001
indentation modulus	*E*_*I*_	10	−0.62	<0.05
cuticle ‘deformation’	*κ*	47	−0.37	<0.01
cuticle ‘strain’	$\hat{\kappa }$	47	0.02	=0.89

In order to assess to which extent the variation of bite force can be explained by changes in muscle volume, a theoretical prediction for the scaling relationship between both is needed. The basis for such a prediction is not obvious, because although the force of a muscle is typically proportional to its cross-sectional area [62], it is *a priori* unclear whether additional volume accumulates in area or length during muscle development. Based on geometric similarity, the cross-sectional area grows in proportion to $V_{m}^{0.67}$, which may provide a reasonable lower bound for the expected scaling relationship. For the upper bound, one may assume that muscle fibre length remains constant, so that all volume accumulates in the cross-section, and muscle force scales in direct proportion to volume; this may be a ‘generous’ upper bound as myofibrils typically grow both in width and in length during muscle maturation [63]. Based on these assumptions, the expected scaling coefficient of bite force lies between two-thirds and one. We observed $F_{b}\propto V_{m}^{1.64}$, in substantial excess of both theoretical predictions (OLS regression on log_10_-transformed data, slope: 1.64, 95% CI: [1.07 | 2.22], *R*^2^ = 0.85; figure 4*a*), suggesting that volume changes alone are insufficient to explain the increase of bite forces.

The mandible closer muscle in callow leaf-cutter ants is thus not only considerably smaller, but may also have a reduced stress capacity. Such an ‘underperformance’ was previously predicted by Muscedere *et al.* [32], who studied the development of cephalic muscles in *Pheidole* ants post eclosion; they found that freshly eclosed callows had muscle fibres that were up to a factor of three thinner, had a non-uniform diameter, and lacked characteristic striation compared to mature ants [32]. Although we were unable to identify the ultrastructure of the muscle from the tomographic scans, we also noticed further developmental differences in addition to changes in volume: muscle fibres of callows were less distinctly separated and often detached from the head capsule (for more details, see electronic supplementary material). The detachment of muscle is surprising, because muscle attachment typically matures in the early steps of muscle morphogenesis, as shown in *Drosophila* (e.g. [63,64]). To test if muscle detachment arose as an artefact of freeze–thawing and sample preparation, we scanned another two callows (≈1 and 3 days old) that underwent the same protocol, but were not used in bite experiments (see electronic supplementary material). Muscle detachment was less severe in these ants, cautiously suggesting that (i) the bite experiment may have caused some muscle fibres to detach and (ii) muscle formation may have only just begun at the point of eclosion; we emphasize that a more thorough study on the muscle morphogenesis in *Atta* is required to draw stronger conclusions.

Substantial muscle development after eclosion appears to be common in insects: the cross-sectional area of beetle flight muscle [65] and locust abdominal muscle [66] increases substantially; flight muscles in bees display a sharp increase of enzyme activity in the first days after eclosion, suggesting biochemical adjustments [38–40]; and during metamorphosis in *Drosophila* flies and *Manduca* moths, the developing adult flight muscles initially lack fully matured motor neurons required for muscle activation (e.g. [67–69]). The ‘underperformance’ of muscle observed in this study is hence likely a result of the combined effects of anatomical, physiological, biochemical and neurological deficiencies. The effect of these deficiencies is rather substantial: a mature worker of 1.5 mg has the same muscle volume as a 4.5 mg callow, but produces four times higher bite forces at a third of its body mass [45,47] (figure 4*a*).

### (b) Flexural rigidity and the mechanical demands on the head capsule during biting

We have demonstrated that callow leaf-cutter ants generate strongly reduced maximum voluntary bite forces, most likely due to incomplete muscle development. Next, we turn our attention to another biomechanical parameter that determines the ability to safely apply large bite forces: the mechanical stability of the head capsule. The ant head capsule is remarkably thin: even for mature ants, it has an average thickness comparable to that of human hair (figure 3*d*), and is only locally reinforced in regions such as the tentorium, the occipital suture, and around the mandibular joints (see below and electronic supplementary material, figure 1*f*,*g*). However, the head capsule has to resist substantial size-specific bite and muscle forces. A single muscle fibre with a diameter of 25−30 μm generates forces up to 0.70 mN, and ants of the considered weight have close to 1000 closer muscle fibres, resulting in a total force of ≈700 mN, more than 10 000 times their body weight [45,47]. To estimate the structural stability of the head capsule, we introduce the flexural rigidity, *D*, the relevant metric for thin plates deformed in bending [70]:

3.1 $$D\propto E_{I}T_{hc}^{3}.$$

Here, *E*_*I*_ is the indentation modulus of the head capsule cuticle, and *T*_*hc*_ is its thickness.

*E*_*I*_ increased significantly with decreasing cuticle brightness (*p* < 0.05; table 1 and figure 3*c*; OLS regression slope: −0.50, 95% CI: [−0.90 | −0.10], *R*^2^ = 0.43). For foragers, the indentation modulus was 7.2 ± 2.1 GPa, 1.5 times higher than for bright callows (*b*_RGB_ > 0.35), 4.9 ± 1.5 GPa; both values are well within the range of moduli reported for insect cuticle (0.4–30 GPa) [71,72]. The relative increase in modulus is consistent with previous work on insects: the bending modulus of locust tibiae increases approximately threefold during the growth phase following the final moult [73]; the storage modulus of beetle elytra increases approximately sixfold in the first week after eclosion [74]; and the tooth hardness of leaf-cutter ant mandibles increases by a factor of nearly three [34], a development associated with increased zinc-concentration and possibly resistance to mandibular wear (also see [42]). In some leaf-cutter ant species, a biomineral layer forms on the epicuticle a week after eclosion, resulting in a twofold increase in hardness [75]. The biomechanical development of insect cuticle is often linked to tanning and sclerotization, a process associated with water loss, cross-linking of cuticle proteins with chitin, and a resulting increase in modulus [50,71,74,76]. Indeed, cuticle hydration affects both indentation modulus and hardness [71,77], and because we conducted experiments of small samples in ambient conditions, we likely overestimate the modulus, and underestimate its increase during post-eclosion development (see fig. 3 in [72] and [74]).

The head capsule thickness, *T*_*hc*_, in turn increased significantly by a factor of two from 16.4 ± 0.4 μm for bright callows to 31.0 ± 5.7 μm for foragers (*p* < 0.001; table 1 and figure 3*d*; OLS regression slope: −0.62, 95% CI: [−0.81 | −0.43], *R*^2^ = 0.87); this variation is comparable to the cuticle thickness range measured from the pronotum of other Myrmicine workers of similar head width (≈1.7 mm, see fig. 2 in [78]). Notably, the increase of the standard deviation with decreasing cuticle brightness suggests a non-uniform increase of cuticle thickness (see figure 3*d*): For bright callows (*b*_RGB_ > 0.35), the standard deviation is 7.8 ± 0.3 μm, significantly smaller than for foragers, 18.9 ± 3.6 μm (Spearman’s rank correlation: *ρ*_8_ = −0.94, *p* < 0.001; this significant difference also holds for the coefficient of variation: *ρ*_8_ = −0.70, *p* < 0.05). The increased variability of the head capsule thickness suggests a ‘targeted’ growth of cuticle during post-eclosion development, perhaps in regions most prone to deformation (also see [79]). Cuticle volume increased significantly, too, in almost direct proportion to cuticle thickness (*p* < 0.05; table 1 and figure 3*b*): Foragers have an average cuticle volume of 0.22 ± 0.09 mm^3^, exceeding that of bright callows by a factor of 2.3 (*b*_RGB_ > 0.35, 0.09 ± 0.02 mm^3^). Cuticle growth post eclosion has been reported for other insects such as locusts [73,80], grasshoppers [81] and moths [82], and is typically associated with the internal deposition of additional layers of endocuticle [73,81]. In the initial growth phase following the final moult, locusts deposit about 1.8 μm of endocuticle per day [73]. Assuming that the age difference between foragers and bright callows is one week, the cuticle growth rate for leaf-cutter ants is about the same, 14.6 μm/7 d ≈ 2 μm/d.

The combined effects of the changes in indentation modulus and cuticle thickness result in a drastic increase of the flexural rigidity with cuticle brightness (figure 4*b*; to obtain a numerical value for *D* from equation (3.1), we used a proportionality constant 1/(12 (1 − 0.3^2^)), see [70]). From the lightest to the darkest workers, the flexural rigidity increased by a staggering factor of 27, from 1.3 to 35.7 mN mm; we note that this result is based on interpolation via regression, and is hence affected by the associated uncertainties of the slope (see CIs in figure 3). The majority of this increase is driven by the change in cuticle thickness (factor of 13.4), which is cubed in equation (3.1), whereas the indentation modulus contributes linearly and thus has a much smaller effect (factor of 2.0). In order to make the implications of this difference in flexural rigidity tangible, we calculated the expected deflection for two plates of the same area, but of different thickness and moduli, approximated by *T*_*hc*_ and *E*_*I*_, respectively, for a forager with a cuticle brightness of *b*_RGB_ = 0.15 and a callow with *b*_RGB_ = 0.5. Under the same load, equal to half of the maximum muscle stress extracted for closely related *A. cephalotes* majors [83], the resulting deflection of callow cuticle is 17 times higher than for dark cuticle; absolute values of deflection are estimated in the electronic supplementary material.

These results invite another hypothesis why callows ‘underperform’ when biting, in addition to continued muscle growth and physiological development (figure 4*a*). Callows may choose to bite with sub-maximal muscle force in order to avoid large deformations of the head capsule. We consider two possible constraints on deformation: (i) absolute deformation, relevant if the elastic deformation is sufficiently large to cause damage inside the head, such as by compressing soft tissues; and (ii), relative deformation, relevant if the stress in the cuticle exceeds its elastic limit, causing fissures or permanent deformation. The absolute cuticle deformation resulting from biting, *κ*, may be estimated from the ratio between maximum bite force and flexural rigidity. For foragers, *κ* = 5.09 ± 1.74 mm^−1^, 1.5 times higher than for bright callows *κ* = 3.36 ± 1.89 mm^−1^. This difference, although significant (*p* < 0.01; table 1), is remarkably small in comparison to the large variation in both bite force and flexural rigidity. The relative cuticle deformation or ‘strain’, in turn, may then be estimated from this absolute deformation as $\hat{\kappa }=\kappa /T_{hc}$. Strikingly, $\hat{\kappa }$ is not significantly correlated with cuticle brightness (*p* = 0.89; table 1 and figure 4*c*). suggesting that maintaining equal cuticle strain may be a constraint on maximum muscle activation. We stress that the numerical values of *κ* and $\hat{\kappa }$ do not equate to actual cuticle deformation and strain, respectively, but are approximately proportional to them; the counterintuitive units arise from the square of a missing length scale that causes the bending moment in the cuticle, which is likely proportional to the constant external head dimensions (also see equation S1 in electronic supplementary material).

Bite forces, muscle ultrastructure and volume, as well as head capsule rigidity all develop in the days following eclosion. Indeed, the variation of all extracted parameters with cuticle brightness follows a similar pattern, with a rapid change across a narrow range of brightness values (figure 3). This mechanical co-development results in an approximately constant mechanical strain in the head capsule cuticle during maximum voluntary bites throughout the callow phase. Future work will need to address to which extent these changes are causally linked, as is observed in vertebrates (e.g. [84–86]).

### (c) Biomechanical limitations of foraging ability in leaf-cutter ants

We set out to investigate if the behavioural transition from within-nest to outside-foraging tasks may arise from variation in biomechanical performance. We have reported evidence for strong changes in maximum voluntary bite force in the days following eclosion. Next, we address briefly if these changes may explain why young callows do not forage. In leaf-cutter ants, foraging involves the cutting and carrying of leaf fragments from fresh vegetation surrounding the nest [43]. The ability of a forager to cut leaves depends on the ratio of two key forces: the maximum bite force, and the minimum force required to initiate and propagate a cut through leaf lamina and veins [87]. Based on published mechanical data for around 1000 tropical leaves, the expected cutting forces range between 7 and 828 mN, with a median of 82 mN [45,61]. Thus, the foragers used in our study would be able to cut approximately half of the measured tropical leaves, whereas bright callows could cut virtually none of them. Foraging also involves the carrying of leaf fragments: A forager of 5 mg may carry fragments of around 15 mg, or three times its body weight [48,88]. This load may sound heavy, but the gravitational force that needs to be overcome is only 0.15 mN, small even compared to the poor bite performance of callows. However, fragments cut from grasses may be as long as 30 mm [48], five times longer than the ants themselves [89] or 20 times their head length [47]; carrying fragments of this length poses a difficult mechanical challenge, which requires continuous adjustments of the neck angle to maintain stability during walking, and likely involves large moments around the neck joint [89,90]. It is unclear if neck muscle develops in concert with the mandible closer muscle; different muscles probably follow different developmental timelines, as shown for mandible closer and antennal muscle in *Pheidole* ants [32]. Whether callows are also less capable of carrying large grass-fragments will thus have to be determined in future work.

Our results suggest that the foraging ability of young workers is largely constrained by a combination of a poor bite performance based on underdeveloped muscles and a low mechanical stability of the head capsule—a finding that probably extends to in-nest cutting as well [91]. We also note that the role of mechanical constraints in influencing age polyethism may continue beyond the time frame considered here: For example, older foragers with worn mandibles may require more than twice the force to cut the same material than those with pristine mandibles [35,92] and are indeed more likely to carry leaves instead of cutting [35]; eventually, the oldest workers often switch to tasks related to waste disposal [23,93].

## 4. Conclusion and outlook

In the days following eclosion, the musculoskeletal bite apparatus of young leaf-cutter ants undergoes substantial biomechanical development: the maximum bite force, muscle volume, head capsule thickness and the cuticle-mechanical properties all increase substantially, from a point where bite forces are too low to cut leaves and the head capsule is too mechanically unstable to resist large muscle forces, to full biomechanical ability. These results suggest that *Atta* leaf-cutter ant callows are not yet able to partake in foraging activities involving cutting—adding direct experimental support to the hypothesis that age polyethism is largely dictated by developmental factors (e.g. [22,24]). The extent to which callows readily perform in-nest tasks such as fungal garden or brood care, in turn, remains poorly understood [17,18]. Future work on the behavioural development of callows inside the nest may be able to integrate the findings of this study to gain a more comprehensive understanding of the behavioural repertoire expansion of leaf-cutter ants. Our findings further suggest exciting avenues for future research on the co-dependency of muscle and cuticle development, which hints at an important role of mechanical stimuli, an area of research that has received considerable interest in vertebrates (e.g. [84–86]), but less so in invertebrates (e.g. [63,94]). Many tasks in colonies of social insects impose mechanical demands, and we thus hope that a biomechanical paradigm will help to increase our general understanding of age polyethism more broadly.

## Data Availability

Raw data are provided in the electronic supplementary material [95]. All analyses are described in the methods section.
