# Correction to: ‘Developmental biomechanics and age polyethism in leaf-cutter ants’ (2023) by Püffel *et al.*

**DOI:** 10.1098/rspb.2023.1983

**Published:** 2023-09-20

**Authors:** Frederik Püffel, Lara Meyer, Natalie Imirzian, Flavio Roces, Richard Johnston, David Labonte

**Keywords:** division of labour, social insects, bite forces, behavioural development

*Proc. R. Soc. B* **290**, 20230355 (Published online 14 June 2023). (doi:10.1098/rspb.2023.0355)

We wish to correct an error in the interpretation of $\hat{\kappa }$, which we used as a proxy for cuticle strain during biting. $\hat{\kappa }$ was calculated as the ratio between absolute cuticle ‘deformation’, *κ*, and the thickness of the head capsule cuticle, *T*_*hc*_. Because strain in thin plates is typically dominated by in-plane deformations, but *κ* estimates out-of-plane deformation, the normalization of *κ* with thickness is probably not an appropriate proxy for cuticle strain. We thus suggest to remove $\hat{\kappa }$ from table 1, and provide an alternative to figure 4*c*—a diagram depicting the variation of *κ* with cuticle brightness. We accordingly suggest the following alternative paragraphs to the last two paragraphs of §3(b), where we now limit the discussion to absolute deformation. This change does not alter any of the fundamental results and conclusions of this work.

## (b) Flexural rigidity and the mechanical demands on the head capsule during biting

These results invite another hypothesis as to why callows ‘underperform’ when biting, in addition to continued muscle growth and physiological development (figure 4*a*). Callows may choose to bite with sub-maximal muscle force in order to avoid large deformations of the head capsule. We approximate the absolute cuticle deformation resulting from biting, *κ*, as the ratio between maximum bite force and flexural rigidity. For foragers, *κ* = 5.09 ± 1.74 mm^−1^, 1.5 times higher than for bright callows, *κ* = 3.36 ± 1.89 mm^−1^. *κ*, although significantly correlated with cuticle brightness (*p* < 0.01; table 1), varies remarkably little in comparison to both bite force and flexural rigidity. We stress that the numerical values of *κ* do not translate to actual cuticle deformations, but are approximately proportional to them; the counterintuitive unit arises from the square of a missing length scale that causes the bending moment in the cuticle, which is likely proportional to the constant external head dimensions (also see equation (S1) in electronic supplementary material).

Bite forces, muscle ultrastructure and volume, as well as head capsule rigidity all develop in the days following eclosion. Indeed, the increase of all extracted parameters with decreasing cuticle brightness follows a similar pattern, with a rapid increase across a narrow range of brightness values (figure 1). This mechanical co-development ensures only a small variation of the mechanical demand on the head capsule cuticle during maximal bites throughout the callow phase. Future work will need to address the extent to which these changes are causally linked, as is observed in vertebrates (e.g. [84–86]).

**Figure 1.**
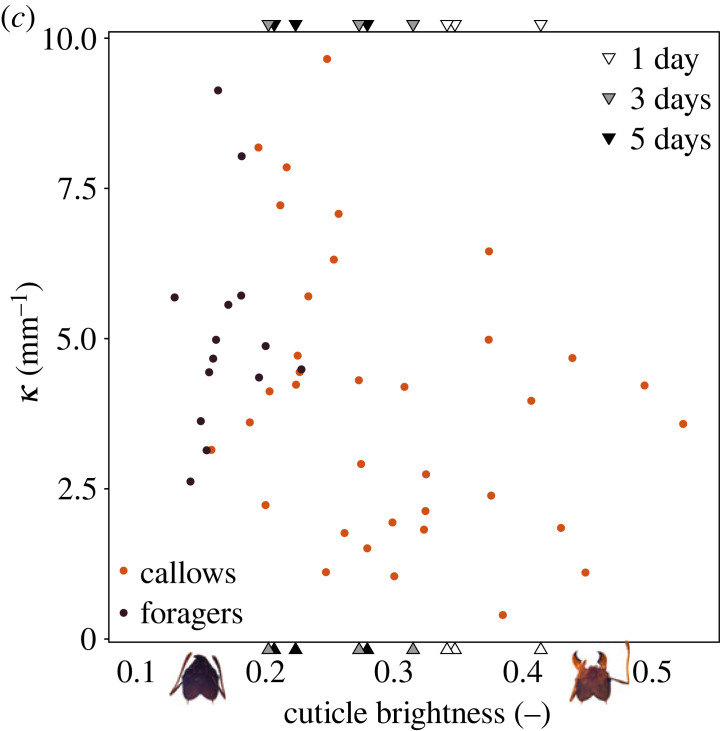
(*c*) The ratio between maximum bite force and flexural rigidity, *κ*, is a proxy for cuticle deformation. *κ* was significantly negatively correlated with cuticle brightness (*p* < 0.01, table 1), but this variation is small compared with the large variation of both bite force and flexural rigidity. The mechanical demand on the head capsule during biting thus likely only varies little across the development post eclosion.

